# Vitamin D endocrine system and osteoclasts

**DOI:** 10.1038/bonekey.2013.229

**Published:** 2014-02-05

**Authors:** Naoyuki Takahashi, Naoyuki Udagawa, Tatsuo Suda

**Affiliations:** 1 Institute for Oral Science, Matsumoto Dental University, Nagano, Japan; 2 Department of Biochemistry, Matsumoto Dental University, Nagano, Japan; 3 Research Center for Genomic Medicine, Saitama Medical University, Saitama, Japan

## Abstract

Vitamin D was discovered as an anti-rachitic agent preventing a failure in bone mineralization, but it is now established that the active form of vitamin D_3_ (1α,25(OH)_2_D_3_) induces bone resorption. Discovery of the receptor activator of nuclear factor -κB ligand (RANKL) uncovered the molecular mechanism by which 1α,25(OH)_2_D_3_ stimulates bone resorption. Treating osteoblastic cells with 1α,25(OH)_2_D_3_ stimulates RANKL expression, which in turn induces osteoclastogenesis. Nevertheless, active vitamin D compounds such as calcitriol (1α,25(OH)_2_D_3_), alfacalcidol (1α(OH)D_3_) and eldecalcitol (1α,25-dihydroxy-2β-(3-hydroxypropoxy) vitamin D_3_) have been used as therapeutic drugs for osteoporosis, as they increase bone mineral density (BMD) in osteoporotic patients. Paradoxically, the increase in BMD is caused by the suppression of bone resorption. Several studies have been performed to elucidate the mechanism by which active vitamin D compounds suppress bone resorption *in vivo*. Our study showed that daily administration of eldecalcitol to mice suppressed neither the number of osteoclast precursors in the bone marrow nor the number of osteoclasts formed in *ex vivo* cultures. Eldecalcitol administration suppressed RANKL expression in osteoblasts. This review discusses how the difference between *in vitro* and *in vivo* effects of active vitamin D compounds on bone resorption is induced.

## Introduction

**Figure 1:**
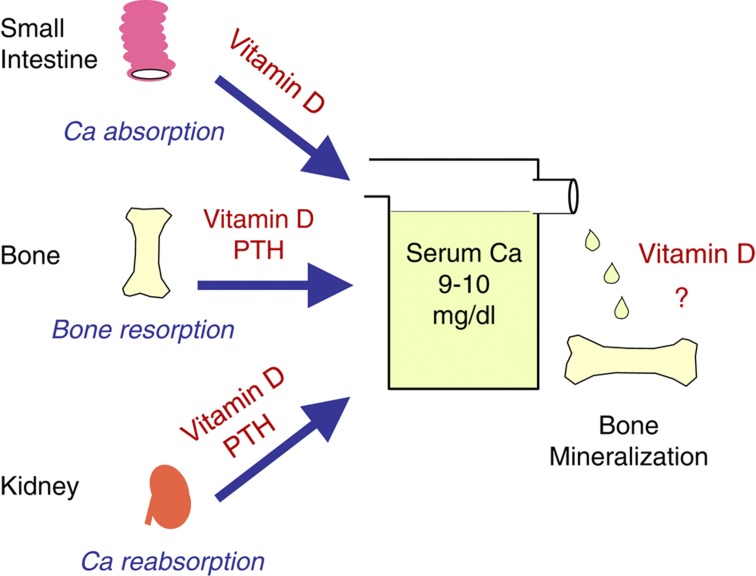
Classical actions of vitamin D to maintain serum calcium homeostasis. Vitamin D is the sole factor that stimulates intestinal calcium absorption. It is proposed that vitamin D and PTH working in concert are necessary to mobilize calcium from the bone and conserve calcium from urine. However, there is no direct evidence that vitamin D directly stimulates osteoblastic bone mineralization.

Vitamin D was discovered as an anti-rachitic agent capable of preventing a failure in bone mineralization. A vitamin D deficiency results in rickets in the young and osteomalacia in adults. The administration of vitamin D to rachitic animals and humans cures impaired bone mineralization. Although vitamin D is the sole factor which stimulates intestinal absorption of calcium, vitamin D and parathyroid hormone (PTH) working in concert are necessary to mobilize calcium from the bone and conserve calcium from urine (Figure 1). Therefore, vitamin D is postulated to directly stimulate osteoblastic bone formation and mineralization; however, there is no evidence to support this proposal.

Vitamin D_3_ is first metabolized to 25-hydroxyvitamin D_3_ (25(OH)D_3_) in the liver and then to 1α,25-dihydroxyvitamin D_3_ (1α,25(OH)_2_D_3_) in the kidney.^1^,^2^ 1α,25(OH)_2_D_3_ is now recognized as a steroid hormone that has a role in maintaining calcium homeostasis through the vitamin D receptor (VDR).^3^ VDR knockout (VDR^−/−^) mice grew normally before weaning.^4^ However, they developed severe hypocalcemia after weaning. Both bone formation and mineralization were severely impaired in VDR^−/−^ mice but were completely recovered by feeding a high-calcium diet.^4^ When the bone isolated from VDR^−/−^ mice was transplanted into wild-type (WT) mice, the VDR^−/−^ bone showed excessive bone formation and mineralization under normocalcemic conditions.^5^ These results suggest that the stimulating effect of 1α,25(OH)_2_D_3_ on bone mineralization is indirect, occurring through stimulation of intestinal calcium absorption.

Carlsson^6^ and Bauer^7^ were the first to realize that a major function of vitamin D in the bone was to induce bone resorption. Using an organ culture system, Raisz *et al*.^8^ showed that 1α,25(OH)_2_D_3_ increased the release of ^45^Ca from the prelabeled bone into the culture medium. When the bone marrow cells were cultured, multinucleated cells having the characteristics of osteoclasts were formed in response to bone-resorbing factors, including 1α,25(OH)_2_D_3_.^9^,^10^,^11^ These results established the concept that 1α,25(OH)_2_D_3_ was an inducer of osteoclastic bone resorption.

Nevertheless, active vitamin D compounds such as calcitriol (1α,25(OH)_2_D_3_), alfacalcidol (1α-hydroxyvitamin D_3_, 1α (OH)D_3_) and eldecalcitol (1α,25-dihydroxy-2β-(3-hydroxypropoxy) vitamin D_3_) have been used as therapeutic drugs to treat osteoporosis in Japan, because they improve bone mineral density (BMD) and decrease the risk of fractures.^12^,^13^,^14^,^15^,^16^ Paradoxically, such beneficial effects are caused by inhibiting osteoclastogenesis. The present review describes the regulation of osteoclast differentiation and possible mechanisms by which active vitamin D compounds suppress bone resorption *in vivo*.

## Structure and function of osteoclasts

**Figure 2:**
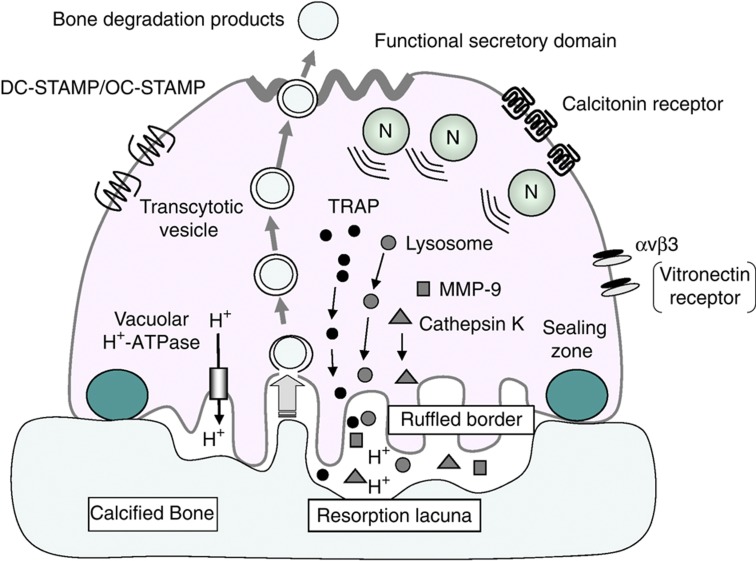
Ultrastructure and function of osteoclasts. Osteoclasts have several characteristics, such as multiple nuclei, abundant mitochondria and a large number of vacuoles and lysosomes. Bone-resorbing osteoclasts form ruffled borders and sealing zones. The resorbing area under the ruffled border is acidic. Vacuolar H^+^-ATPase localized in the ruffled border is involved in the transport of protons into the resorption lacunae. Enzymes such as cathepsin K, MMP9 and TRAP are secreted into the resorption lacuna to degrade bone matrix proteins. Matrix degradation products are endocytosed from the central portion of the ruffled border, packaged into transcytotic vesicles and secreted from the functional secretory domain. Osteoclasts express large numbers of calcitonin receptors and α_v_β_3_ vitronectin receptors. Osteoclasts also express DC-STAMP and OC-STAMP, which are involved in the cell–cell fusion of osteoclasts.

Osteoclasts have several characteristics, including multiple nuclei, abundant mitochondria and a large number of vacuoles and lysosomes (Figure 2).^17^,^18^ The most characteristic feature of bone-resorbing osteoclasts is the presence of ruffled borders and sealing zones (also called clear zones). The sealing zone attaches osteoclasts to the bone surface and isolates resorption lacunae from the surroundings. Resorption lacunae under the ruffled border are acidic, which favors the dissolution of bone minerals. Vacuolar H^+^-ATPase is localized in the ruffled border membranes. The transport of protons into resorption lacunae is mediated by vacuolar H^+^-ATPase. Lysosomal enzymes are also secreted into lacunae to degrade the organic matrix in the bone. Tartrate resistant-acid phosphatase (TRAP) is highly expressed in osteoclasts, and some TRAP is secreted into resorption lacunae. Histochemical TRAP staining is widely used to identify osteoclasts *in vivo* and *in vitro*. Some matrix degradation products are endocytosed from the central portion of the ruffled border domain, incorporated into transcytotic vesicles and exocytosed through the functional secretory domain in the basolateral membrane.^18^ Osteoclasts express calcitonin receptors. Calcitonin suppresses bone-resorbing activity of osteoclasts. Osteoclasts also express the vitronectin receptor, α_v_β_3_ integrin, which is involved in attachment to the bone matrix. Recently, Fuller *et al*.^19^ reported that α_v_β_3_ integrins were not only necessary but also sufficient for the induction of resorptive behavior in osteoclasts.

Multinucleated osteoclasts are formed by cell–cell fusion of mononuclear preosteoclasts. The dendritic cell-specific transmembrane protein (DC-STAMP), a seven-transmembrane protein, was first identified as a protein responsible for the fusion of preosteoclasts.^20^,^21^ No multinucleated osteoclasts were observed, but many preosteoclasts were detected in DC-STAMP^−/−^ mice. The bone-resorbing activity of DC-STAMP^−/−^ preosteoclasts was lower than that in multinucleated osteoclasts. DC-STAMP^−/−^ mice develop mild osteopetrosis. Miyamoto *et al*.^22^ recently reported that osteoclast-stimulatory transmembrane protein (OC-STAMP), another seven-transmembrane protein, was also involved in the fusion of preosteoclasts. OC-STAMP^−/−^ mice exhibited a complete lack of cell–cell fusion of preosteoclasts, although the expression of DC-STAMP was normal in these cells. These results suggest that the fusion of osteoclasts is regulated by both OC-STAMP and DC-STAMP.

## Regulation of osteoclast differentiation by osteoblastic cells

In 1981, Rodan and Martin^23^ proposed that osteoblasts may intervene in the process of osteoclastic bone resorption. Their argument was based on observations that osteoblasts, but not osteoclasts, expressed receptors of bone-resorbing factors, such as PTH and prostaglandin E_2_ (PGE_2_). Bone-lining cells were also suggested to be involved in the regulation of osteoclastic bone resorption.^24^ Based on this concept, we established a mouse co-culture system of calvarial osteoblastic cells and splenocytes to investigate osteoclastogenesis. Osteoclast-like multinucleated cells were formed in this co-culture in response to bone-resorbing factors, including 1α,25(OH)_2_D_3_, PTH and PGE_2_.^25^ No osteoclasts were formed when cell-to-cell contact between osteoblastic cells and splenocytes was inhibited by a membrane filter. These results suggest that microenvironments provided by osteoblastic cells support the osteoclastic differentiation of splenic precursors.

Experiments with the osteopetrotic *op/op* mice have established the role of macrophage colony-stimulating factor (M-CSF) for osteoclastogenesis. An extra thymidine insertion in the M-CSF gene was found in *op/op* mice, which generated a stop codon in the downstream,^26^ suggesting that *op/op* mice could not produce the active M-CSF protein. The administration of recombinant M-CSF to *op/op* mice restored impaired bone resorption.^27^ Osteoblastic cells obtained from *op/op* mice could not support osteoclastogenesis in co-cultures with WT splenocytes.^28^ The addition of M-CSF to the co-culture with *op/op* osteoblastic cells induced osteoclast formation from WT splenocytes in response to 1α,25(OH)_2_D_3_. In contrast, *op/op* splenic precursors differentiated into osteoclasts when co-cultured with WT osteoblastic cells. M-CSF was shown to be involved not only in the proliferation of osteoclast precursors but also in their differentiation into osteoclasts.^29^

In 1992, we proposed a hypothesis for the mechanism of osteoclastogenesis: bone-resorbing factors act on osteoblastic cells to induce a membrane-bound factor, named ‘osteoclast differentiation factor (ODF)'.^30^ Osteoclast precursors express ODF receptors, recognize ODF through cell–cell interaction with osteoblastic cells and differentiate into osteoclasts in the presence of M-CSF. Chambers *et al*.^31^ reported a similar factor ‘stromal cell-derived osteoclast forming activity'.

## Discovery of the RANKL-RANK interaction for osteoclastogenesis

**Figure 3:**
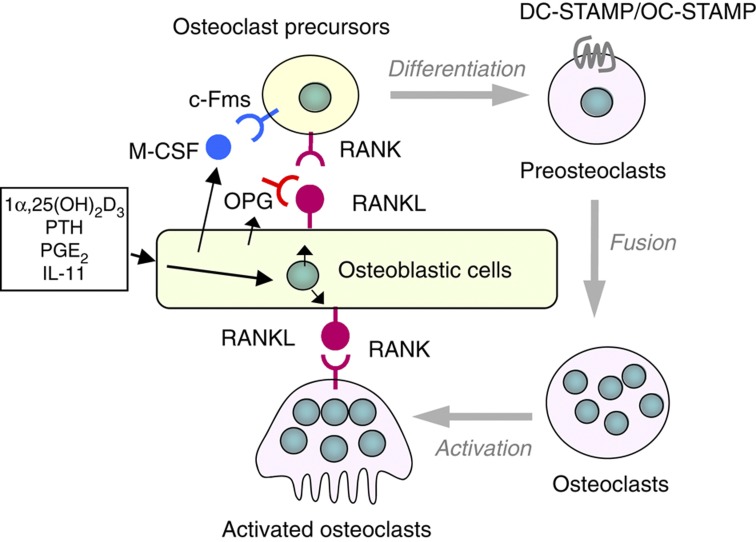
Regulation of osteoclast differentiation and function by osteoblastic cells. Bone resorption-stimulating factors act on osteoblastic cells to induce the expression of RANKL as a membrane-associated factor. Osteoblastic cells constitutively produce M-CSF. Osteoclast precursors express receptors RANK and c-Fms and differentiate into osteoclasts in the presence of RANKL and M-CSF. Osteoblastic cells secrete OPG, which inhibits the RANKL–RANK interaction between osteoblastic cells and osteoclast precursors. Multinucleated osteoclasts also express RANK, and RANKL induces the bone-resorbing activity of osteoclasts via the interaction with RANK.

Osteoprotegerin (OPG; also called osteoclastogenesis inhibitory factor (OCIF)) was cloned as a member of the tumour necrosis factor (TNF) receptor family in 1997.^32^,^33^ This protein lacked a transmembrane domain but had an N-terminal signal peptide for a secreted protein. OPG/OCIF suppressed osteoclast formation in co-cultures treated with bone-resorbing factors.^32^ Therefore, OPG/OCIF was speculated to act as a decoy receptor for ODF. Using OPG/OCIF as a probe, the ODF cDNA was isolated from a library of the bone marrow stromal cell line ST2.^34^ ODF is a transmembrane protein of the TNF ligand family, and its expression in osteoblastic cells was upregulated by bone-resorbing factors. Lacey *et al*.^35^ also cloned an OPG ligand (OPGL), which was identical to ODF. Molecular cloning of ODF/OPGL demonstrated that ODF/OPGL was identical to TNF-related activation-induced cytokine (TRANCE)^36^ and receptor activator of nuclear factor (NF)-κB ligand (RANKL),^37^ which had been identified by other groups. The receptor of ODF was confirmed to be receptor activator of NF-κB (RANK). Thus, ODF, OPGL, TRANCE and RANKL are different names for the same ligand, and OPG and OCIF are different names for the same decoy receptor.^38^,^39^ According to the President's Committee on Nomenclature (2000) of the American Society for Bone and Mineral Research, the terms ‘RANKL' as the ligand, ‘RANK' as the receptor and ‘OPG' as the decoy receptor are used in text (Figure 3).

Osteoblastic cells express RANKL as a membrane-associated factor in response to bone-resorbing factors.^38^ 1α,25(OH)_2_D_3_ is one of the most potent inducers of RANKL in osteoblastic cells. VDR^−/−^ osteoblastic cells failed to support 1α,25(OH)_2_D_3_-induced osteoclastogenesis in co-cultures with WT splenocytes.^40^ The molecular mechanism by which 1α,25(OH)_2_D_3_ enhances transcription of the RANKL gene is described by Pike *et al*.^41^

## NFATc1 as a master transcription factor for osteoclastogenesis

**Figure 4:**
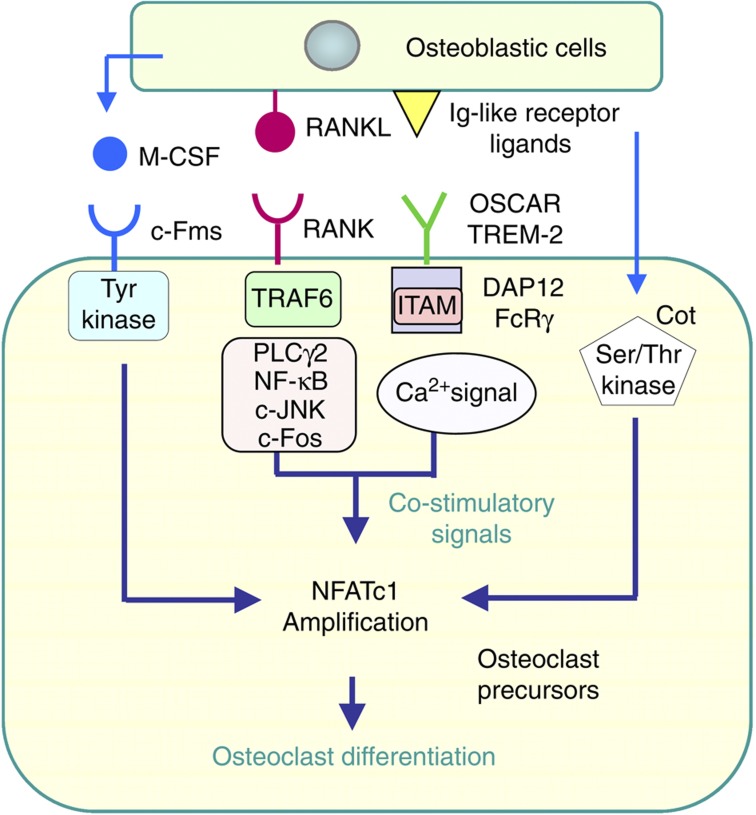
Intracellular signals in osteoclast precursors induced by osteoblastic cells. Osteoclast differentiation is induced by amplifying NFATc1, a master transcription factor for osteoclastogenesis. The M-CSF receptor, c-Fms, has a tyrosine kinase domain in the cytoplasmic region, and tyrosine kinase-mediated signals regulate the proliferation and differentiation of osteoclast precursors. The binding of RANKL to RANK results in the recruitment of TRAF6, which activates PLCγ, MAP kinases, NF-κB and AP1 (c-Fos/c-Jun). Immunoglobulin-like receptors, TREM2 and OSCAR, are associated with ITAM-containing DAP12 and FcRγ, respectively. Osteoblastic cells express the ligands of immunoglobulin-like receptors. RANK and ITAM signaling lead to Ca^2+^ oscillations, which induce the amplification of NFATc1. NFATc1 is also activated in a Ca^2+^ oscillation-independent, but Cot kinase-dependent, manner. Cot is activated by the cell–cell interaction with osteoblastic cells.

Signaling pathways required for osteoclastogenesis have been identified since the discovery of RANKL (Figure 4).^40^,^42^,^43^,^44^ Binding of RANKL to RANK triggers TNF receptor-associated factor 6 (TRAF6)-dependent signaling, which activates phospholipase Cγ (PLCγ), mitogen-activated protein (MAP) kinases and NF-κB. RANKL-induced osteoclastogenesis is also dependent on co-stimulatory signaling through immunoreceptor tyrosine-based activation motif (ITAM)-containing adaptors, Fc receptor common γ (FcR γ) and DNAX-activating protein of 12 kDa (DAP12).^45^ FcR γ associates with osteoclast-associated receptor (OSCAR), while DAP12 associates with triggering receptor expressed on myeloid cells 2 (TREM-2). RANK-mediated and ITAM-mediated signals cooperate to further activate PLCγ, which generates inositol-1,4,5-triphosphate (IP_3_). IP_3_ then induces Ca^2+^ mobilization from the endoplasmic reticulum through IP_3_ receptors and generates Ca^2+^ oscillations. Ca^2+^ oscillations contribute to the amplification of NF of activated T-cells, cytoplasmic 1 (NFATc1), the master transcription factor for osteoclastogenesis.^44^ Because ITAM-mediated signaling is crucial for the robust induction of NFATc1, this pathway is called ‘co-stimulatory signaling' for RANK-induced osteoclastogenesis. Osteoblastic cells have been proposed to express ligands for OSCAR and TREM-2. OSCAR was recently shown to bind to a specific motif of collagen.^46^

NFATc1 is also activated by a Ca^2+^ oscillation-independent pathway (Figure 3).^47^ This pathway, as well as the ITAM pathway, is activated by osteobalstic cells. FK506, an inhibitor of calcineurin, suppresses Ca^2+^ oscillations in osteoclast precursors. NFATc1 concentrations in osteoclast precursors were increased in co-cultures with osteoblastic cells even in the presence of FK506. Osteoclast precursors derived from IP_3_ receptors type 2 and type 3 double knockout mice, in which RANKL-induced Ca^2+^ oscillations were absent, normally differentiated into osteoclasts in co-culture with WT osteoblastic cells.^47^ Cot (cancer osaka thyroid) serine/threonine kinase in osteoclast precursors was activated by cell–cell interactions with osteoblastic cells.^48^ The activation of Cot in osteoclast precursors increased NFATc1 protein levels through phosphorylation-dependent protein stabilization. These results suggest that NFATc1 amplification is induced by both Ca^2+^ oscillation-dependent and -independent pathways. Mice doubly deficient in DAP12 and FcR γ have been shown to exhibit severe osteopetrosis owing to the lack of osteoclasts.^45^ These results suggest that the ITAM signal is physiologically important in controlling osteoclast differentiation.

## Characteristics of osteoclast precursors *in vivo*

**Figure 5:**
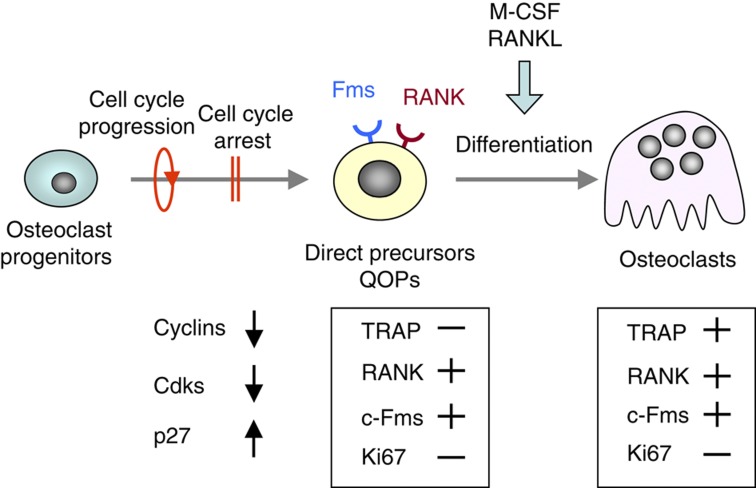
Two cell cycle-related events in osteoclastogenesis. Cell cycle progression and subsequent withdrawal in osteoclast progenitors are required for their differentiation into osteoclasts *in vitro*. The direct osteoclast precursors have been named ‘cell cycle-arrested quiescent osteoclast precursors (QOP)'. The cell cycle arrest in QOP is induced by the disappearance of cyclins and Cdks, and the appearance of p27^KIP1^. Osteoclasts express TRAP, RANK and c-Fms, but not Ki67, while QOP express RANK and c-Fms but not TRAP or Ki67.

Attempts to identify osteoclast precursors *in vivo* have established a model for osteoclastogenesis. Mizoguchi *et al*.^49^ reported that cell cycle progression and subsequent cell cycle arrest in osteoclast progenitors were required for their differentiation into direct osteoclast precursors (Figure 5). The expression of cyclins and cyclin-dependent kinases (Cdks) was suppressed, whereas that of p27^KIP1^, a Cdk inhibitor, was upregulated in the precursors during their differentiation into osteoclasts.^49^ Neither these precursors nor osteoclasts expressed Ki67, a cell proliferation marker. Therefore, these osteoclast precursors were named ‘cell cycle-arrested quiescent osteoclast precursors' (QOPs). QOPs, but not osteoclasts, exist in RANKL^−/−^ mice and *op/op* mice. Bromodeoxyuridine (BrdU) is a nucleoside analog that is incorporated into dividing nuclei. RANKL^−/−^ mice were given BrdU in their drinking water and were injected with RANKL for 2 days. Osteoclasts appeared in the bone of RANKL^−/−^ mice in response to the RANKL injection. More than 70% of nuclei in RANKL-induced osteoclasts were negative for BrdU.^49^ *op/op* mice were also given BrdU and were injected with M-CSF for 7 days. Many osteoclasts appeared in the bone in *op/op* mice injected with M-CSF. More than 80% of nuclei in those osteoclasts were BrdU negative.^49^ These results suggest that osteoclasts are formed from QOPs in both RANKL^−/−^ mice and *op/op* mice.

QOPs are expected to express RANK and c-Fms, but not TRAP or Ki67. The distribution of QOPs was examined in RANKL^−/−^ mice using these markers, because QOPs, but not osteoclasts, exist in these mice. RANK and c-Fms-double positive (Fms^+^/RANK^+^) cells were detected along the surface of trabecular bones in RANKL^−/−^ mice.^49^ They were negative for Ki67. QOPs were detected near alkaline phosphatase-positive (ALP^+^) osteoblasts, suggesting that osteoblasts support the presence of QOPs in the bone.

QOPs were isolated as RANK-positive (RANK^+^) cells from the WT bone marrow. RANK^+^ cells expressed c-Fms but not macrophage-associated markers, such as F4/80 and CD11b. Bone marrow-derived QOPs showed no phagocytic activity and did not proliferate in response to M-CSF.^50^ They could not differentiate into dendritic cells but differentiated into osteoclasts. These results suggest that QOPs are committed precursors of osteoclasts.

## Circulating osteoclast precursors

**Figure 6:**
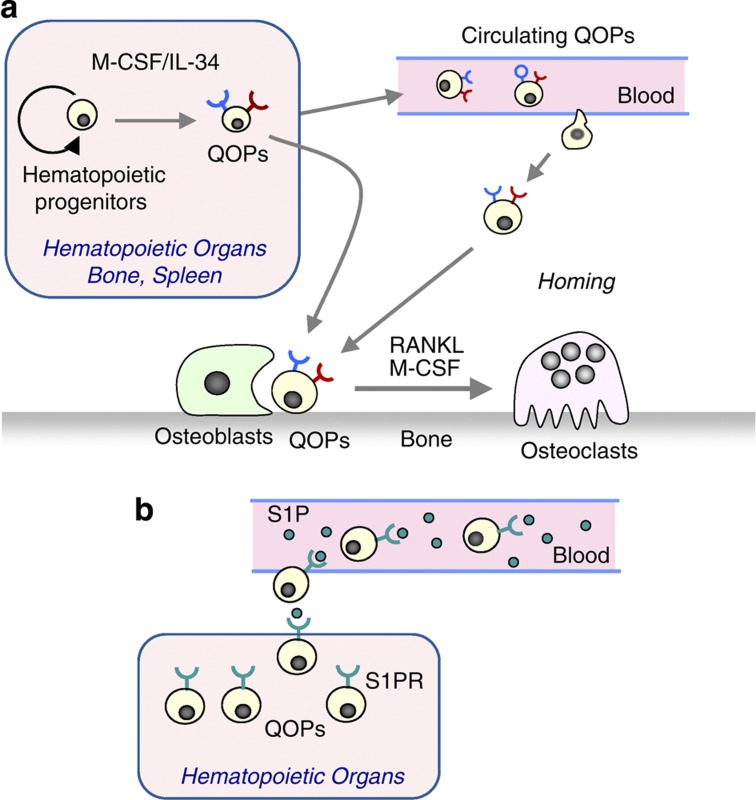
A hypothetical model for osteoclastogenesis *in vivo*. (**a**) QOPs are generated from hematopoietic progenitors in hematopoietic organs, such as the bone marrow and spleen. M-CSF and/or IL-34 are involved in the generation of QOPs. Some QOPs circulate in the blood. Osteoblasts may have a role in homing of QOPs to the bone. (**b**) S1P in the blood controls the trafficking of QOPs from hematopoietic tissues to the blood through S1P receptors (S1PR). Administration of FYT720, an S1P agonist, promotes the egress of QOPs from hematopoietic tissues into bloodstream.

Some QOPs are circulating. Collagen disks containing bone morphogenetic protein 2 (BMP-2) were implanted into WT mice. These mice were given BrdU. After implantation for 2 weeks, osteoclasts were detected in ectopic bone tissues induced by BMP-2. Most nuclei in these osteoclasts were BrdU negative.^50^ Osteoclasts were not induced in control collagen disks. Collagen disks containing BMP-2 were also implanted into RANKL^−/−^ mice. QOPs appeared in BMP-2-induced bone tissues but not in the control disks in RANKL^−/−^ mice. When RANKL was injected into these RANKL^−/−^ mice, osteoclasts appeared in BMP-2-containing disks but not in the control disk.^51^ These results suggested that some QOPs circulated in the blood and settled in the bone (Figure 6a). The distribution of QOPs was similar to that of ALP^+^ osteoblasts in RANKL^−/−^ mice,^49^ suggesting that ALP^+^ bone-forming osteoblasts are somehow involved in homing of QOPs to the bone. Using fluorescent imaging techniques, Kotani *et al*.^52^ showed that some osteoclasts were generated from circulating precursors. At present, it is not known that all QOPs found on the bone surfaces are derived from circulating QOPs.

Recent studies suggest that c-Fms-mediated signaling is required for the differentiation of hematopoietic progenitors into QOPs. Interleukin 34 (IL-34) is a newly discovered ligand for c-Fms.^53^ The amino-acid sequence of IL-34 is different from that of M-CSF; however, the biological activity of the two ligands is similar. IL-34 is predominantly expressed in the spleen and not in bone, while M-CSF is expressed in both the bone and spleen. c-Fms^+^/RANK^+^ QOPs were not detected in the bone but were detected in the spleen of *op/op* mice.^54^ This suggested that QOPs moved from the spleen to the bone in response to M-CSF injections. Indeed, removal of the spleen (splenectomy) blocked M-CSF-induced osteoclastogenesis in *op/op* mice. The expression of RANK in precursors is shown to be induced by M-CSF.^55^,^56^ These results suggest that IL-34 has a pivotal role in maintaining splenic QOPs in *op/op* mice and that some QOPs circulate in the blood. On the basis of these findings, we proposed a model of osteoclast formation *in vivo* (Figure 6a). M-CSF/IL-34 may be required for the differentiation of hematopoietic progenitors into QOPs. Some QOPs circulate in the blood and settle in the bone. Osteoblasts appear to be involved in homing of QOPs to the bone.

Sphingosine-1-phosphate (S1P), a lipid mediator enriched in the blood, was shown to control the dynamic migration of osteoclast precursors between the blood and bone. Ishii *et al*.^57^ reported that FYT720, an agonist of S1P, increased circulating osteoclast precursors and regulates bone homeostasis. We also confirmed that the injection of FTY720 to mice increased circulating QOPs in the blood.^50^ These results suggest that the interaction between S1P and S1P receptors (SIPR) is involved in the egress of QOPs from hematopoietic tissues into the blood (Figure 6b).

## Effects of the *in vivo* administration of vitamin D compounds on bone metabolism

**Figure 7:**
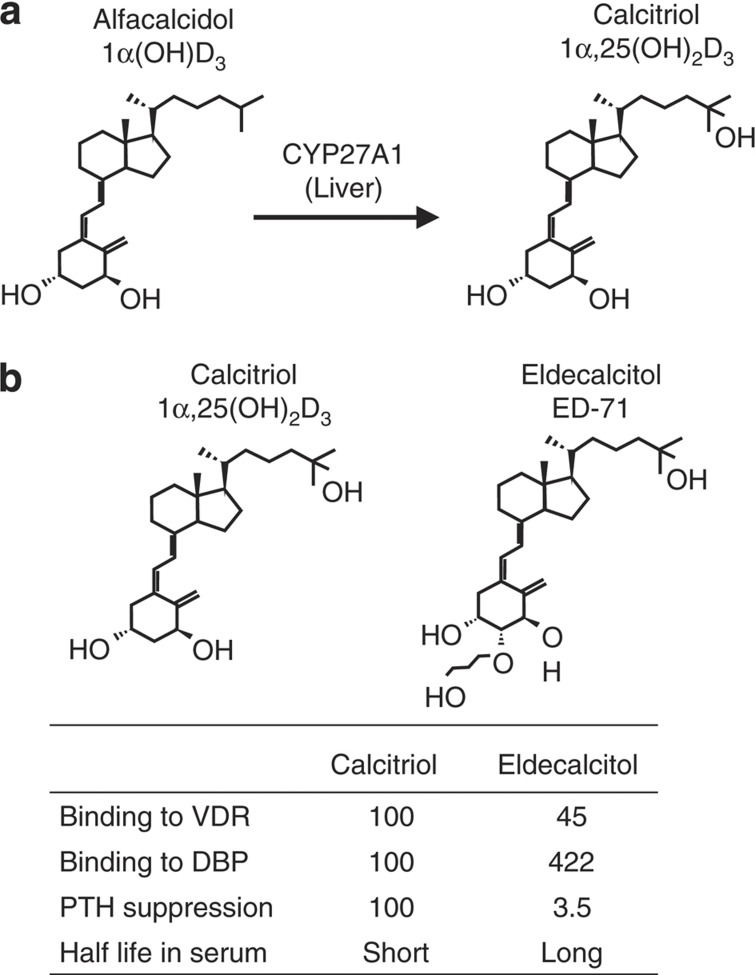
Structures and characteristics of vitamin D compounds used as therapeutic drugs for osteoporosis. (**a**) Alfacalcidol, a prodrug of calcitriol (1α,25(OH)_2_D_3_), is metabolized to calcitriol by liver CYP27A1. Alfacalcidol, therefore, acts as calcitriol *in vivo*. (**b**) Eldecalcitol has a hydroxypropoxy substituent at the 2β position. Eldecalcitol is not metabolized to calcitriol *in vivo*. Some characteristics of eldecalcitol are compared with those of calcitriol.

Calcitriol, alfacalcidol and eldecalcitol have been used as therapeutic drugs for the treatment of osteoporosis in Japan. Alfacalcidol is rapidly converted to calcitriol by liver CYP27A1 (Figure 7a). Therefore, alfacalcidol is believed to act as calcitriol *in vivo*. Eldecalcitol was isolated from vitamin D analogs based on the activity that stimulated BMD *in vivo*^58^ (Figure 7b). A clinical study showed that eldecalcitol increased BMD more effectively than alfacalcidol.^16^ Eldecalcitol binds more weakly to VDR but more strongly to serum vitamin D binding protein (DBP) than calcitriol.^59^ Eldecalcitol more weakly suppresses PTH production than calcitriol. The half-life of eldecalcitol in serum is longer that than that of calcitriol.^60^ Such differences may be related to the higher efficacy of eldecalcitol than alfacalcidol.

The active form of vitamin D enhances intestinal calcium absorption, leading to an increase in serum calcium levels and suppression of bone resorption. 1α,25(OH)_2_D_3_ at pharmacological concentrations may also inhibit PTH secretion and stimulate calcium renal calcium re-absorption. Integrating such significant changes may suppress osteoclastic bone resorption. Recent data also indicate that autocrine/paracrine activities of vitamin D are detected in each of the major bone cell types where CYP27B1 [25(OH)D_3_ 1α-hyrdoxylase] is expressed.^61^ Therefore, it is likely that 1α,25(OH)_2_D_3_ produced by bone cells is also involved in the suppression of bone resorption in an autocrine/paracrine manner.

Clinical studies have shown that daily administration of eldecalcitol increases lumbar and hip BMD in osteoporotic patients with vitamin D supplementation.^14^,^16^ Transnet hypercalcemia was observed in some patients, but none of them sustained hypercalcemia. Both bone resorption and formation were suppressed by the administration of eldecalcitol, suggesting the existence of a coupling mechanism between bone resorption and bone formation. We therefore speculated that daily administration of active vitamin D compounds may directly suppress bone resorption.

**Figure 8:**
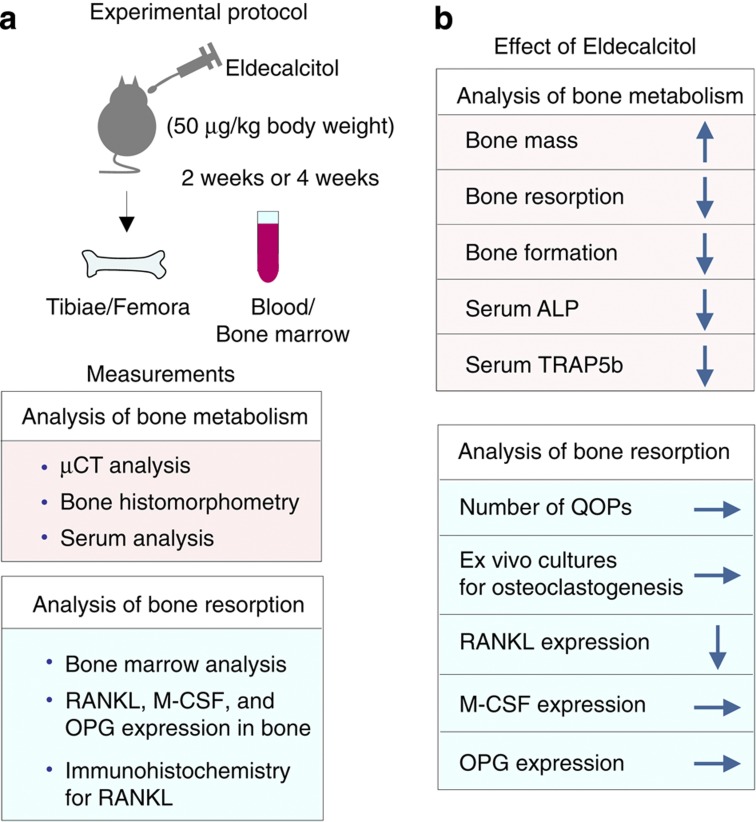
Effects of the daily administration of eldecalcitol on bone metabolism in mice. (**a**) Protocol of experiments. Eldecalcitol (50 ng per kg body weight) or vehicle was administered daily to 9-week-old male mice for 2 and 4 weeks. Bone samples and serum were recovered for analyses of bone metabolism. (**b**) Results obtained. The daily administration of eldecalcitol increased bone mass with the suppression of both bone resorption and bone formation. The suppression of bone resorption was more prominent than that of bone formation. Neither the number of QOPs nor the differentiation potential of QOPs into osteoclasts was affected by eldecalcitol administration. Eldecalcitol administration suppressed RANKL mRNA expression but not M-CSF or OPG mRNA expression in tibiae. Immunohistochemistry confirmed that the number of RANKL^+^ cells in trabecular bones was decreased by eldecalcitol administration.

We proposed two possible explanations for the inhibitory action of active vitamin D compounds on bone resorption *in vivo*. First, long-term exposure to pharmacological concentrations of active vitamin D compounds may alter the number of osteoclast precursors, including QOPs. The second possibility is that daily administration may alter the microenvironment of the bone, which supports osteoclastogenesis. We then examined the effects of the daily administration of eldecalcitol on osteoclastogenesis in mice^62^ (Figure 8). Treating mice with eldecalcitol increased BMD in the metaphysis of femurs. Bone and serum analyses showed that both bone resorption and formation were suppressed by eldecalcitol administration, suggesting that the eldecalcitol-induced increase in BMD was due to the suppression of bone resorption.^62^

We then examined how eldecalcitol suppressed bone resorption *in vivo*^62^ (Figure 8). Eldecalcitol administration failed to affect the number of QOPs in the bone marrow. F4/80^+^ and CD11b^+^ cells in the bone marrow are believed to be precursors of QOPs. The populations of F4/80^+^ and CD11b^+^ cells in the bone marrow remained unchanged under the eldecalcitol-treated condition. An *ex vivo* culture showed that osteoclast formation from QOPs was not affected by eldecalcitol administration. We examined the second possibility that eldecalcitol may alter the microenvironment for osteoclastogenesis (Figure 8). Eldecalcitol administration significantly suppressed RANKL mRNA expression but not M-CSF or OPG mRNA expression in tibiae. RANKL^+^ cells were immunohistochemically observed as a line in the hypertrophic cartilage area and around trabecular and cortical bones. The distribution of RANKL^+^ cells was evaluated as RANKL-positive cell surface (RANKLS). Eldecalcitol administration significantly decreased RANKLS preferentially around trabecular bones.^62^ Bone loss in ovariectomized mice is an animal model of postmenopausal osteoporosis. The daily administration of eldecalcitol as well as calcitriol to ovariectomized mice suppressed RANK L expression in the bone and increased BMD. Eldecalcitol more effectively inhibited bone resorption than calcitriol *in vivo*.^62^ These results suggest that the daily administration of active vitamin D compounds changes the bone microenvironments in osteoporotic patients.

## Why does the daily administration of active vitamin D compounds suppress bone resorption?

There are several possible explanations for the suppression of bone resorption by active vitamin D compounds *in vivo*. The *in vivo* administration of active vitamin D compounds may directly affect osteoblastic cells to suppress RANKL expression. However, this possibility is unlikely, because the suppression of RANKL expression by active vitamin D compounds has never been reported *in vitro*. The *in vivo* administration of large amounts of active vitamin D compounds to WT mice always induces osteoclastic bone resorption.

**Figure 9:**
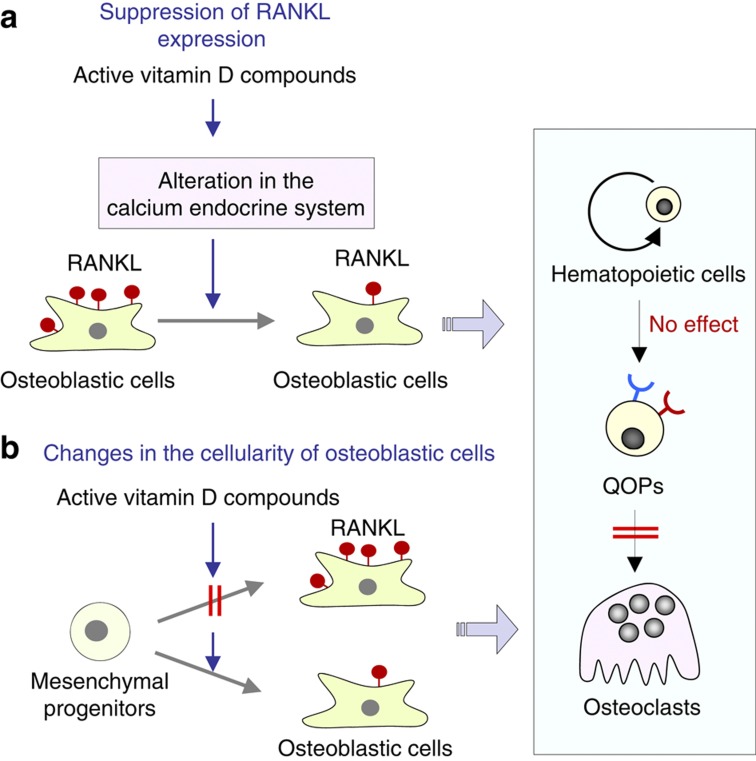
Two potential mechanisms for the *in vivo* effects of active vitamin D compounds in inhibiting bone resorption. (**a**) Long-term exposure to active vitamin D compounds may influence the calcium endocrine system, which suppresses RANKL expression in osteoblastic cells in an integrated manner. (**b**) Daily administration of active vitamin D compounds may decrease RANKL activity in osteoblastic cells or induce a population shift of osteoblastic cells (changes in the cellularity of osteoblastic cells).

**Figure 10:**
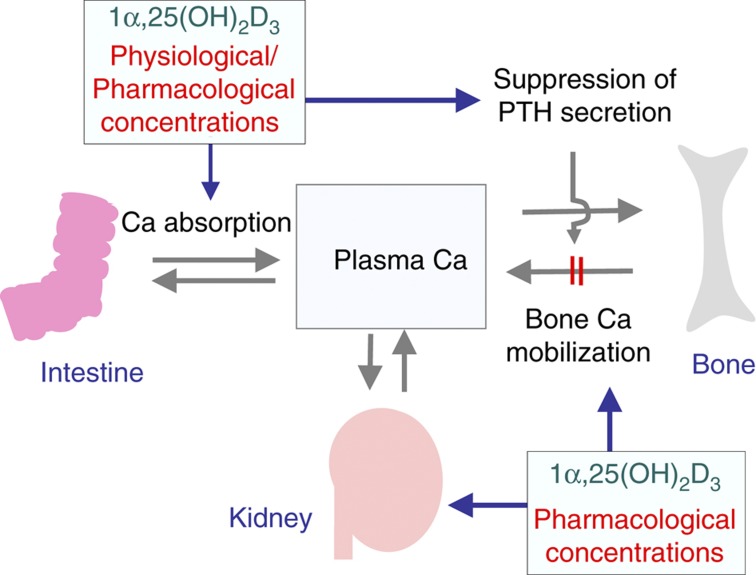
A hypothetical concept for the calcium endocrine system controlled by pharmacological concentrations of 1α,25(OH)_2_D_3_. 1α,25(OH)_2_D_3_ at physiological and pharmacological concentrations stimulates intestinal calcium absorption and inhibits PTH secretion. 1α,25(OH)_2_D_3_ at pharmacological concentrations may inhibit PTH secretion and stimulate calcium renal calcium re-absorption. Integrating such significant changes may suppress RANKL expression in osteoblastic cells.

We believe that this phenomenon is induced by long-term exposure to pharmacological concentrations of active vitamin D compounds. Two potential mechanisms are conceivable for the *in vivo* reduction in RANKL expression in the bone (Figure 9). One possible mechanism is as follows: pharmacological concentrations of active vitamin D compounds in serum may alter the calcium endocrine system through intestine of parathyroid glands. Such an alteration in the calcium endocrine system may create favorable circumstances for the suppression of RANKL expression in osteoblastic cells (Figure 9a). Using intestinal-specific VDR^−/−^ mice, Lieben *et al*.^63^ showed that maintaining normocalcemic has priority over skeletal integrity. Daily administration of active vitamin D compounds may suppress bone resorption to maintain normocalcemia. Active vitamin D compounds may also cause small but significant changes in the calcium endocrine system *in vivo*. Integrating such changes may suppress RANKL expression in osteoblastic cells (Figure 10).

The other possible mechanism is related to osteoblast differentiation: Daily administration of active vitamin D compounds may influence the differentiation of mesenchymal progenitors into osteoblastic cells, resulting in the suppression of RANKL expression (Figure 9b). RANKL is expressed by most osteoblast-lineage cells such as the bone marrow stromal cells, osteoblasts and osteocytes. RANKL was reported to be expressed preferentially by immature osteoblasts, and expression levels decreased during osteoblast maturation.^64^ Another study showed that osteocytes more effectively supported osteoclast formation in a co-culture than mature osteoblasts.^65^ de Freitas *et al*.^66^ reported that the daily administration of eldecalcitol to ovariectomized rats suppressed bone resorption and stimulated preosteoblasts to differentiate into mature osteoblasts *in vivo*. Gardiner *et al*.^67^ reported that transgenic mice overexpressing VDR in mature cells in the osteoblastic lineage showed increased bone formation and decreased bone resorption in mice. The transgene effects on bone formation and bone resorption exhibited site specificity of bone tissues. These results suggest that active vitamin D compounds directly act on osteoblastic cells to suppress bone resorption in a site-specific manner. These findings also suggest that active vitamin D compounds may decrease RANKL activity in osteoblastic cells on the trabecular bone surface or may induce a population shift of osteoblastic cells, resulting in a decrease in the number of RANKL-expressing osteoblasts.

The direct action of active vitamin D compounds on osteoclast precursors has also been proposed to explain its suppressive effect on osteoclastogenesis. 1α,25(OH)_2_D_3_ inhibits RANKL-induced osteoclastic differentiation in cultures of osteoclast precursors in the absence of osteoblastic cells. 1α,25(OH)_2_D_3_ suppressed the expression of c-Fos, a transcription factor essential for osteoclastogenesis, in osteoclast precursors.^68^ 1α,25(OH)_2_D_3_ was also shown to stimulate the expression of interferon β, an inhibitor of osteoclastogenesis, in osteoclast precursors.^69^ Kikuta *et al*.^70^ demonstrated that calcitriol and eldecalcitol inhibited bone resorption by modulating the S1P receptor system. The inhibitory effects of active vitamin D compounds on osteoclast formation have been observed at concentrations higher than 10^−9^ M in these *in vitro* experiments. Therefore, mechanisms other than the direct action of active vitamin D compounds on osteoclast precursors may be involved in active vitamin D-induced suppression of osteoclastogenesis *in vivo*.

## Conclusion

Active vitamin D compounds stimulate osteoclast formation in a co-culture of osteoblastic cells and hematopoietic cells. Osteoblastic cells express RANKL in response to 1α,25(OH)_2_D_3_. Therefore, 1α,25(OH)_2_D_3_ has been believed to stimulate osteoclastic bone resorption. However, active vitamin D compounds are used as therapeutic drugs for osteoporosis, because they increase BMD *in vivo* due to the suppression of bone resorption. Thus, the effects of active vitamin D compounds on bone resorption *in vitro* and *in vivo* are opposite. We investigated the mechanism by which active vitamin D compounds inhibited bone resorption *in vivo*. QOPs were identified as the direct precursors of osteoclasts *in vivo*. Daily administration of eldecalcitol did not affect the generation of QOPs but suppressed RANKL expression in osteoblasts. Several possible explanations exist for the suppression of RANKL in osteoblasts by active vitamin D compounds *in vivo*. Pharmacological concentrations of active vitamin D compounds in serum may alter the calcium endocrine system, which may create circumstances for the suppression of RANKL expression in osteoblasts. An alternative possibility is the direct action of vitamin D on the bone: active vitamin D compounds may affect the cellularity of the osteoblast lineage. As a result, the number of RANKL-positive osteoblasts decreases. The direct action of active vitamin D compounds on osteoclast precursors has also been proposed to explain their suppressive effects on osteoclastogenesis. Further studies using osteoblast-specific VDR-deficient mice and also osteoclast precursor-specific VDR-deficient mice will elucidate the discrepancy observed between the *in vitro* and *in vivo* effects of active vitamin D compounds on bone resorption. Such experiments are currently being conducted in our laboratories.
